# Effects of resistin on porcine ovarian follicle steroidogenesis in prepubertal animals: an *in vitro* study

**DOI:** 10.1186/1477-7827-11-45

**Published:** 2013-05-17

**Authors:** Agnieszka Rak-Mardyła, Martyna Durak, Ewa Łucja Gregoraszczuk

**Affiliations:** 1 Department of Physiology and Toxicology of Reproduction, Institute of Zoology, Jagiellonian University in Cracow, Gronostajowa 9, Cracow, 30-387, Poland

**Keywords:** Resistin, Prepubertal porcine ovarian follicle, Steroidogenesis

## Abstract

**Background:**

Resistin was first reported to be an adipocyte-specific hormone, but recent studies have indicated a connection between resistin and reproductive function. However, it is not yet known if resistin is expressed by the ovary and if it can affect steroidogenesis in ovarian follicles from prepubertal pigs.

**Methods:**

In this study, using real time PCR, immunoblotting, and ELISA, we quantified resistin expression and concentration in maturing ovarian follicles (small, 3–4 mm; medium, 4–5 mm; large, 6–7 mm) collected from prepubertal pigs. In addition, the dose-responsive effects of recombinant human resistin (0.1, 1, 10, and 100 ng/ml) on steroid hormone (i.e., progesterone [P4], androstendione [A4], testosterone [T], and estradiol [E2]) secretion in culture medium and steroidogenic enzyme (i.e., CYP11A1, 3betaHSD, CYP17A1, 17betaHSD, and CYP19A1) expression in ovarian follicles were determined.

**Results:**

We observed that resistin gene and protein expression increased significantly (*P* < 0.05) during follicular growth, with large follicles expressing the highest level of this adipokine. Recombinant resistin also increased P4, A4, and T secretion by up-regulating the steady state levels of CYP11A1, 3betaHSD, CYP17A1, and 17betaHSD. Recombinant resistin had no effects on E2 secretion and CYP19A1 expression in ovarian follicles.

**Conclusion:**

Our results show resistin expression in ovarian follicles from prepubertal pigs for the first time. We also show that recombinant resistin stimulates steroidogenesis in ovarian follicles by increasing the expression of CYP11A1, 3betaHSD, CYP17A1, and 17betaHSD. The presence of resistin in the porcine ovary and its direct effects on steroidogenesis suggest that resistin is a new regulator of ovary function in prepubertal animals.

## Background

Adipose tissue is known to control many cellular events via endocrine, paracrine, or autocrine mechanisms. Adipose tissue is rich in adipokines, secreted cytokines, and hormones that participate in several physiological and pathological processes such as food intake and metabolic control, diabetes, atherosclerosis, immunity, and reproductive function [1-3]. Leptin, adiponectin, and resistin are three well studied adipokines. Of these, resistin is a cysteine-rich C-terminal domain protein belonging to the resistin-like molecule (RELM) family [4]. In humans, resistin is expressed largely by adipocytes [5], and its serum level is elevated in morbidly obese individuals [6]. While the role of resistin in energy homeostasis is still unclear, previous studies have shown it to exhibit changes in expression that are similar to those of leptin [7,8]. For instance, fasting reduced both resistin and leptin levels, and feeding resulted in their concomitant increase [4,8]. Other studies have suggested a connection amongst resistin, obesity, and insulin resistance [9,10], illustrating the importance of adipokines in mammalian physiology.

In recent studies, many investigators have linked resistin to reproductive function. Nogueiras et al. [11] showed resistin to increase both basal and human chorionic gonadotropin (hCG)-stimulated testosterone secretion dose dependently in rat testis organ cultures *in vitro*. They also demonstrated resistin expression in the testis to be regulated by luteinizing hormone (LH) and follicle stimulating hormone (FSH), two pituitary hormones. In female rats, resistin expression was up-regulated in adipose tissue from pubertal animals [12]. Interestingly, resistin expression in the pituitary gland was found to be regulated by nutritional-, age-, and gender-specific factors, and its expression was highest in the pituitary gland of prepubertal mice [13]. Furthermore, resistin expression in adipocytes was 2-fold higher in women with polycystic ovarian syndrome (PCOS) than in healthy women [14]. Dafopoulus et al. [15] also demonstrated that serum resistin levels remained unchanged in normal cycling women, illustrating that resistin secretion from adipocytes is unaffected by fluctuations in sex steroid levels.

Recently, some data have suggested that resistin could effect on ovary function. Maillard et al. [16] demonstrated resistin expression in bovine and rat ovaries, and showed that resistin modulate granulosa cells function such as steroidogenesis and proliferation, in basal state or in response to IGF-I *in vitro*. This is in agreement with a study by Jones et al. [17], which reported resistin expression throughout the rat estrous cycle. Study Spicer et al. [18] showed that resistin inhibits steroidogenesis of undifferentiated (small follicles) granulosa cells and inhibits mitogenesis of differentiated (large follicles) granulosa cells collected from cattle. In human cultured theca cells, recombinant resistin triggered 17α-hydroxylase activity, a marker of ovarian hyperandrogenism in women with PCOS [19].

Considering data of Morash et al., 2002 [13] showing increasing resistin mRNA expression in pituitary of prepubertal mice and their suggestions that resistin could have functional implications during prepubertal time we hypothesis that resistin could be important local modulator of ovarian function during prepubertal time. To our knowledge, there is no study reporting the expression of resistin in ovarian follicles from prepubertal pigs or the effects of resistin on ovarian steroidogenesis. In the present study, we 1) investigate steady state resistin expression and protein levels in developing ovarian follicles collected from prepubertal pigs, 2) quantify resistin concentrations in these ovarian tissues, and 3) study the effects of recombinant human resistin on ovarian steroidogenesis by investigating the levels of different steroid hormones secretion and steroidogenic enzymes expression.

## Methods

### Reagents

M199 medium and phosphate buffered saline (PBS) were purchased from CytoGen, Poland. Antibiotic antimycotic solution (100×), fetal bovine serum (FBS, heat inactivated), TRIS, sodium deoxycholate, Nonidet NP-40, sodium dodecyl sulfate (SDS), protease inhibitor (EDTA-free), dithiothreitol (DTT), Tween 20, and bromophenol blue were obtained from Sigma-Aldrich (St. Louis, MO, USA). Recombinant human resistin was obtained from Phoenix Pharmaceuticals, Inc. (Burlingame, CA, USA). Human resistin was utilized in this experiment because porcine resistin was not readily available at the onset of the experiment. Human resistin differs from porcine resistin only by one amino acids [20].

### Sample collection

Porcine ovaries were collected from prepubertal (4–5 months of age) crossbred gilts (Large White and Polish Landrace) at a local abattoir. Approximately 15 min elapsed from slaughter to ovary collection. Small (SF, 3–4 mm; *n* = 6), medium (MF, 4–5 mm; *n* = 6) and large (LF, 6–7 mm; *n* = 6) follicles were obtained from animals, as described previously [21]. For each experiment described below, six ovaries from three different animals were selected. Since each ovary yielded four to six follicles, the total number of follicles for each preparation varied between 24 and 36. This approach was used to minimize experimental variation throughout the study. Follicular fluid was also aspirated from follicles using a sterile needle and syringe.

### Experimental procedure

In *Experiment 1* analysis of resistin gene expression in ovarian follicles was determined. For RNA isolation, whole ovarian follicles after excision of ovary and follicular fluid collected, were immediately frozen in liquid nitrogen and then stored at -70°C.

*Experiment 2* quantified resistin expression in whole ovarian follicles, and determined resistin concentrations protein in follicular fluid. SFs, MFs, and LFs were homogenized twice in ice-cold lysis buffer (50 mM Tris–HCl (pH 7.5) at 22°C containing 100 mM NaCl, 0.5% sodium deoxycholate [wt/vol], 0.5% NP-40 [vol/vol], 0.5% SDS [wt/vol], and protease inhibitor). Lysates were cleared by centrifugation at 15,000 × *g* at 4°C for 30 min, and protein content was determined by a protein assay (Bio-Rad Laboratories, Munchen, Germany) using bovine serum albumin (BSA) as a standard. All samples were stored at -20°C until further analysis.

*Experiment 3* investigated the short-term effects of recombinant human resistin on steroid hormone (i.e., P4, A4, T, and E2) secretion and steroidogenic enzyme (i.e., CYP11A1, 3βHSD, CYP17A1, 17βHSD, and CYP19A1) expression. After isolation, follicles were cut, using small scissors, to facilitate penetration of the compounds into the tissue and removal oocytes and follicular fluids. Whole ovarian follicles, including theca and granulosa cells were cut to small pieces (2–3 mm) and individually cultured in 24-well plates with M199 medium supplemented with 5% FBS [vol/vol] and increasing concentrations of resistin (0.1, 1, 10, and 100 ng/ml) at 37°C in a humidified atmosphere containing 5% CO_2_ [vol/vol]. These doses were chosen based on a previously published study [12] and on *Experiment 2*. After 24 h, conditioned culture media were collected and stored at -20°C for steroid hormone determination. Cultured ovarian follicles was homogenized twice in ice-cold lysis buffer and centrifuged at 15,000 × *g* at 4°C for 30 min. Protein content was determined as described above. All samples were stored at -20°C until further analysis. It should be noted that this *in vitro* model was previously used to study the function of leptin in the porcine ovary [22]. Based on our experience, this model mimics ovarian physiology more closely than co-cultures of different ovarian cells or cell lines.

*Experiment 4* examined the short-term effects of recombinant resistin on steroidogenic enzyme (i.e., *CYP11A1*, *HSD3B1* [*3βHSD*], *CYP17A1*, *HSD17B1* [*17βHSD*], and *CYP19A1*) mRNA expression. This experiment was similar to *Experiment 3*, except that cultured ovarian follicles was frozen immediately in liquid nitrogen and then stored at -70°C for RNA isolation.

### Total RNA isolation and cDNA synthesis

Isolation of total RNA, including a 15 min treatment with DNase I, was carried out with the High Pure RNA Tissue kit (Roche Applied Science, Mannheim, Germany). RNA concentrations were determined by spectrophotometry at 260 and 280 nm (BioPhotometer Plus, Eppendorf, Germany), and 1 μg of RNA was used for reverse transcription. Reverse transcription was performed using the Transcriptor First Strand cDNA Synthesis kit (Roche Applied Science) in combination with 50 pmol/μL anchored-oligo(dT)_18_ primer and 600 pmol/μL random hexamer primers. The reverse transcription reaction was incubated at 25°C for 10 min. This was followed by two subsequent incubations at 50°C for 60 min and 85°C for 5 min, and then cooling to 4°C. Samples were stored at -20°C until further analysis.

### Real time PCR

Real time PCR analyses were performed using either StepOne Real-Time PCR (Applied Biosystems, Carlsbad, CA, USA) or LightCycler®Nano SW1.0 Real-Time PCR (Roche Diagnostics, Indianapolis, IN, USA) systems. *Taq*Man Gene Expression assays were used to quantify mRNA expression (Table 1). GAPDH was used as an internal control. Quantitative PCR was performed with 100 ng cDNA, 1 μL *Taq*Man Gene Expression primers, and 10 μL *Taq*Man PCR master mix (Applied Biosystems) in a final reaction volume of 20 μL. After a 2 min incubation at 50°C, thermal cycling conditions were 10 min at 95°C, followed by 40 cycles of 15 sec at 95°C and 1 min at 60°C to determine the cycle threshold number (*C*_t_) for quantitative measurement. Data were analyzed using the comparative threshold cycle (Ct) method and expressed as a ratio of target gene to GAPDH.

**Table 1:** Characteristic of investigated gens

**Gene symbol**	**Gene name**	**Catalog number**	**Reference sequence**
*RETN*	resistin	Ss03381389_u1	NM_213783.1
*CYP11A1*	cytochrome P450, family 11, subfamily A, polypeptide 1	Ss03384849_u1	NM_214427.1
*HSD3B1 (3βHSD)*	hydroxy-delta-5-steroid dehydrogenase, 3 beta- and steroid delta-isomerase 1	Ss03391752_m1	NM_001004049.1
*CYP17A1 (CYP17)*	cytochrome P450 17A1	Ss03394947_m1	NM_214428.1
*HSD17B1 (17βHSD)*	hydroxysteroid (17-beta) dehydrogenase 1	Ss04245959_g1	NM_001128472.1
*CYP19A1 (CYP19)*	cytochrome P450 19A1	Ss03384876_u1	NM_214429.1
*GAPDH*	glyceraldehyde-3-phosphate dehydrogenase	Ss03375629_u1	NM_001206359.1

*TaqMan* gene expression assays were used to quantify mRNA expression.

### Immunoblot analysis

Immunoblotting was performed as previously described [21]. Briefly, equal amounts of lysate (~30–50 μg protein/lane) were separated on 10–15% *T* SDS-polyacrylamide gels. Separated proteins were transferred onto nitrocellulose membranes and blocked with 5% non-fat dry milk in Tris-buffered saline containing 0.1% Tween-20 [vol/vol] (TBS-T). Membranes were then incubated overnight at 4°C with anti-resistin (cat. #sc-17575), anti-CYP11A1 (cat. #sc-18040), anti-3βHSD (cat. #sc-30820), anti-CYP17A1 (cat. #sc-46084), anti-17βHSD (cat. #sc-26963), or anti-CYP19A1 (cat. #sc-14244) antibodies (1:200, Santa Cruz Biotechnology, Inc., Santa Cruz, CA, USA) on a shaker. After washing with TBS-T, membranes were incubated with a horseradish peroxidase-conjugated secondary antibody (Santa Cruz Biotechnology, Inc.). Immune complexes were detected by chemiluminescence (ECL) using Western Blotting Luminol reagent (Santa Cruz Biotechnology, Inc.) and visualized with a Chemidoc™ XRS^+^ system (Bio-Rad Laboratories). Proteins of interest were quantified using Image Lab™ 2.0 software (Bio-Rad Laboratories). An anti-β-actin antibody (1:3000) was used as a loading control (Cat. #A5316, Sigma-Aldrich).

### Resistin ELISA

A commercially available human resistin enzyme-linked immunosorbent assay (ELISA) (cat. # EIA-4945, DRG International, Inc., Mountainside, NJ, USA) was used to quantify resistin concentrations in ovarian follicles lysates and follicular fluid. Samples were run in triplicate within the same assay. The sensitivity of the resistin assay was 0.016–1 ng/ml, and the intra- and inter-experimental coefficients of variation were 10% and 15%, respectively.

### Hormone ELISAs

Steroid hormone (i.e., P4, A4, T, and E2) levels were determined in conditioned culture media using commercially available ELISA kits (cat. #s EIA-1561, EIA-3265, EIA-1559, and EIA-2693, DRG Diagnostic, Marburg, Germany). E2 levels were also measured in follicular fluid, and samples were run in duplicate within this assay. The sensitivity of each assay was 0.045 ng/ml for P4, 0.019–10 ng/ml for A4, 0.083–16 ng/ml for T, and 9.7–2000 pg/ml for E2. The intra- and inter-experimental coefficients of variation were 6.99% and 4.34% for P4, 5.6% and 12.1% for A4, 3.28% and 6.71% for T, and 2.71% and 6.72% for E2, respectively. Cross-reactivity between T and A4 was 0.9%, and between T and E2 was <0.1%. Cross-reactivity amongst E2, A4, and T was 0%, but cross-reactivity with estrone was 0.2% based on specification sheets provided by the manufacturer. All other samples were run in quadruplicate within each assay.

### Statistical analysis

Real time PCR, Western blot and ELISA experiments were performed three independent times. In each experiment six ovaries from three different animals were selected. Data were plotted as mean ± S.E.M. All remaining data were shown as the mean ± S.D. Statistical analyses were performed using GraphPad Prism 5 software (La Jolla, CA, USA). Data were analyzed using a one-way analysis of variance (ANOVA) test, followed by Tukey’s honestly significant difference (HSD) test. Statistical significance is indicated by different letters or * *P* < 0.05.

## Results

### Resistin expression and concentration in ovarian follicles

**Figure 1:**
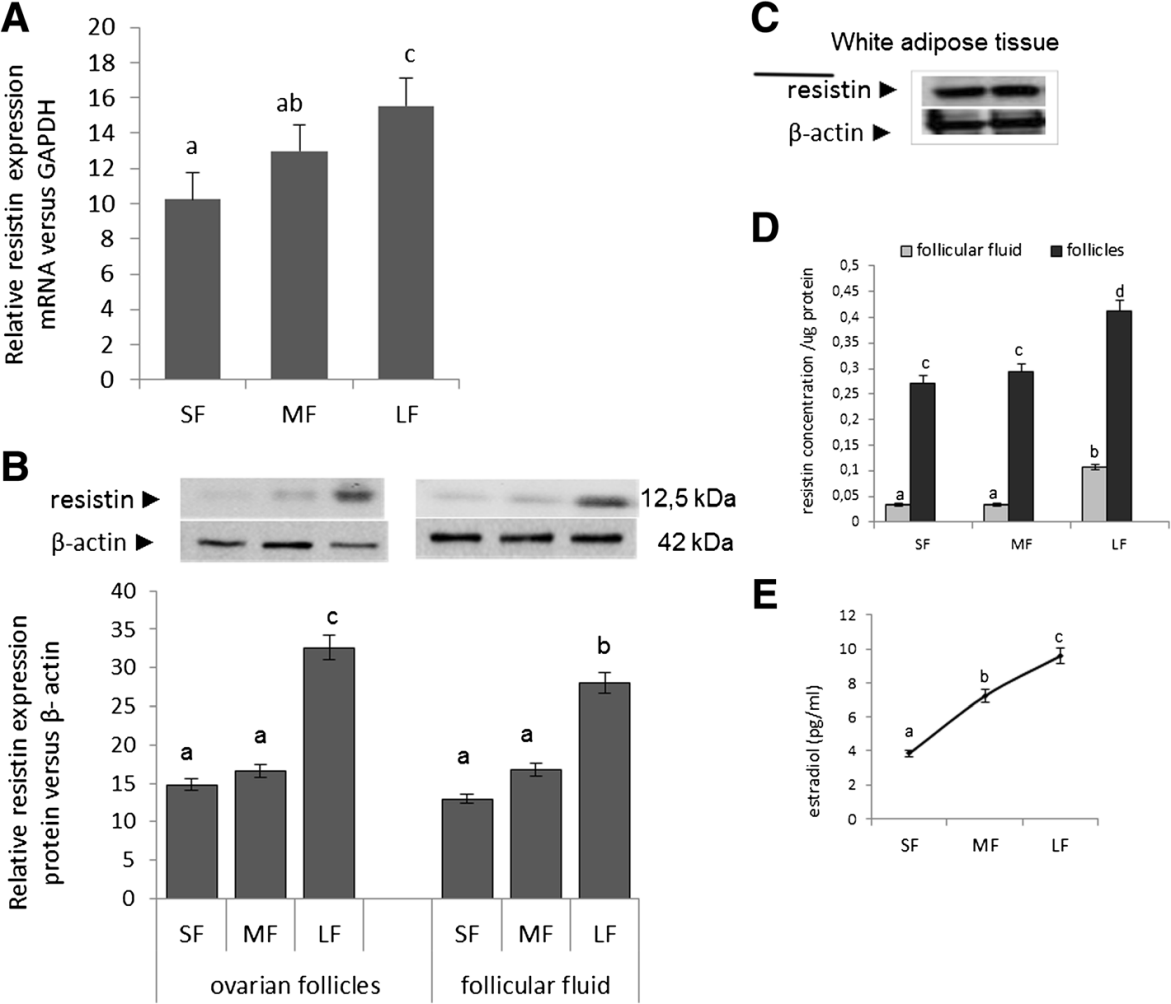
**Resistin expression and concentration in ovarian follicles.** Resistin expression and concentration in ovarian follicles collected from prepubertal animals; **A**) The ratio of resistin gene intensity to GAPDH intensity was used to represent the abundance of resistin mRNA in the ovarian follicles. **B**) The amount of protein (50 μg) in every sample was checked by immunoblot using anti-β-actin antibody. Upper panels: representative blots of tissue homogenates resolved by 15% SDS–PAGE, immunoblotted with resistin antibodies and reprobed with β-actin antiserum. Lower panels: results of densitometric quantification of bands. The data are expressed as the mean±SEM of rations relative to GAPDH or to β-actin. **C**) For positive control we used porcine white adipose tissue. **D**) Concentrations of resistin in follicular fluid and ovarian tissue. Different letters indicate statistically significant differences among groups (*P* < 0.05). **E**) Estradiol concentrations in follicular fluid.

Real time PCR experiments showed that ovarian follicles obtained from prepubertal pigs expressed resistin. Resistin expression in ovarian follicles increased significantly with follicular growth, with LFs expressing the highest level of this adipokine (1.5-fold increase compared to SFs and 1.2-fold increase compared to MFs, *P* < 0.05) (Figure 1).

To confirm real time PCR results, immunoblotting was performed using SF, MF, and LF lysates and follicular fluid. The steady state level of resistin increased significantly in maturing follicles as well as in follicular fluid (Figure 1B). In agreement with the data shown in Figure 1A, resistin was highest in both LF lysate and follicular fluid (2-fold for SFs and 1.8-fold for MFs in follicular fluid, and 1.8 fold to SFs and 1.6 fold to MFs in ovarian follicles tissue) (*P* < 0.05). For positive control we used porcine white adipose tissue (Figure 1C).

Resistin levels were higher in SFs (0.27 ng/μg protein), MFs (0.29 ng/μl protein), and LFs (0.411 ng/μg protein) than in follicular fluid collected from corresponding follicles (0.03, 0.03, and 0.106 ng/μg protein, respectively) (Figure 1D, *P* < 0.05). The increase in resistin during follicular growth also correlated with a steady increase in estradiol in follicular fluid collected from SFs, MFs, and LFs (3.87, 7.22, and 9.58 pg/ml, respectively) (Figure 1E).

### Effects of resistin on the secretion of steroid hormones

**Figure 2:**
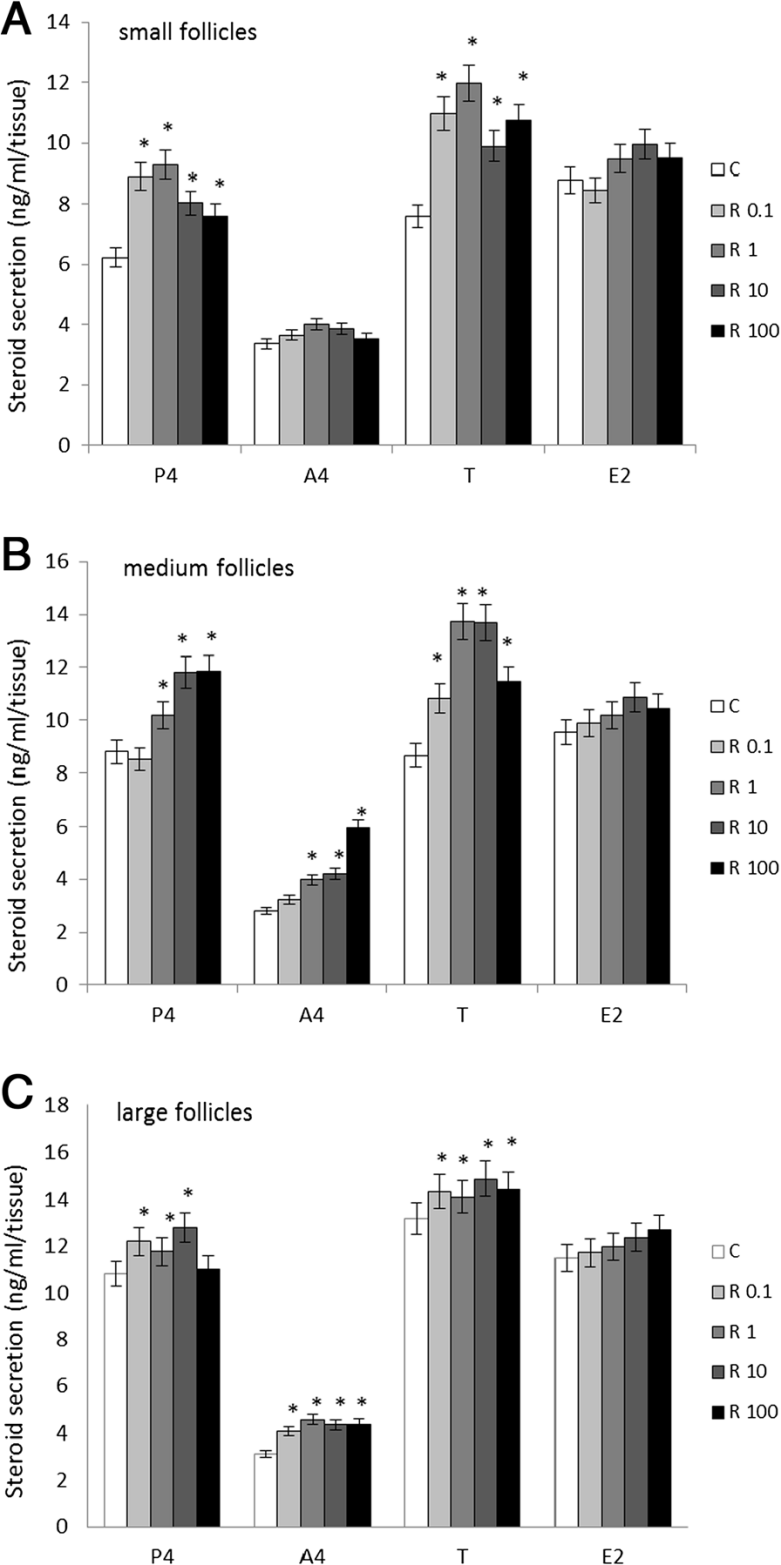
**Effects of recombinant human resistin (0.1, 1, 10, and 100 ng/ml) on steroid hormone (i.e., progesterone [P4], androstendione [A4], testosterone [T], and estradiol [E2]) secretion in culture medium from A) small, B) medium and C) large ovarian follicles.** ELISA experiments were performed three independent times. In each experiment six ovaries from three different animals were selected. Data were plotted as mean±S.E.M. Different letters indicate statistically significant differences among groups (*P* < 0.05).

Basal steroid secretion in conditioned culture media collected from SFs was 6.2 ng/ml for P4, 3.3 ng/ml for A4, 7.5 ng/ml for T, and 8.7 ng/ml for E2. Recombinant resistin at increasing concentrations stimulated P4 (8.9, 9.2, 8, and 7.6 ng/ml at 0.1, 1, 10, and 100 ng/ml doses of resistin, respectively) and T (10.9, 11.9, 9.8, and 10.7 ng/ml at 0.1, 1, 10 and 100 ng/ml doses of resistin, respectively) secretion. Both A4 and E2 in SF conditioned culture media remained unchanged by recombinant resistin (Figure 2A, *P* < 0.05).

Basal steroid secretion in MF conditioned culture media was 8.7 ng/ml for P4, 2.7 ng/ml for A4, 8.6 ng/ml for T, and 9.5 ng/ml for E2. Recombinant resistin at increasing concentrations stimulated P4 (10.1, 11.8, and 11.8 ng/ml, respectively), A4 (3.9, 4.2, and 5.9 ng/ml, respectively), and T (10.8, 13.7, 13.6, and 11.4 ng/ml, respectively) secretion. Recombinant resistin had no effect on E2 secretion (Figure 2B; *P* < 0.05).

Finally, basal steroid secretion in LF conditioned culture media was 10.8 ng/ml for P4, 3.1 ng/ml for A4, 13.1 ng/ml for T, and 11.4 ng/ml for E2. Recombinant resistin at increasing concentrations up-regulated P4 (12.2, 11.7, and 12.8 ng/ml, respectively), A4 (4, 4.5, 4.3, and 4.3 ng/ml, respectively), and T (14.3, 14.1, 14.8, and 15.9 ng/ml, respectively) secretion. Likewise, E2 secretion remained unchanged by recombinant resistin in LF conditioned culture media (Figure 2C, *P* < 0.05).

### Effects of recombinant human resistin on the mRNA expression of steroidogenic enzymes

**Figure 3:**
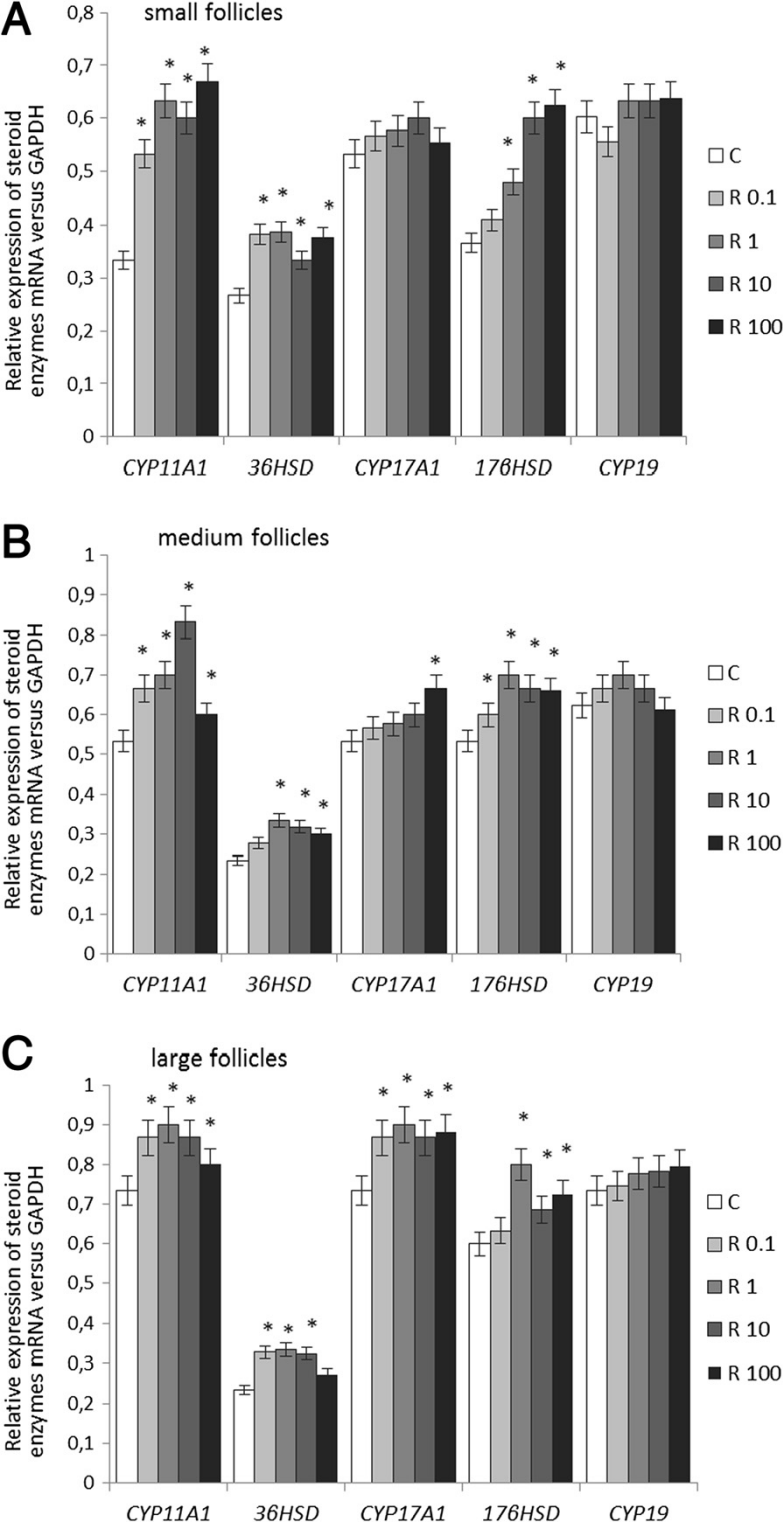
**Effects of recombinant human resistin (0.1, 1, 10, and 100 ng/ml) on the mRNA expression of steroidogenic enzymes (i.e., *CYP11A1, 3βHSD, CYP17A1, 17βHSD, *and *CYP19A1*) in A) small, B) medium and C) large ovarian follicles.** The expression of mRNA has been determined by real time-PCR. The expression of each gene was normalized to the expression of GAPDH. Real time PCR experiments were performed three independent times. In each experiment six ovaries from three different animals were selected. Data were plotted as mean±S.E.M. Statistical significance is indicated by * *P* < 0.05.

To investigate the effects of increasing concentrations of recombinant resistin (0.1, 1, 10, and 100 ng/ml) on steroidogenic enzyme mRNA expression, real time PCR was performed on total RNAs isolated from SFs, MFs, and LFs. The expression of each gene (i.e., *CYP11A1*, *3βHSD*, *CYP17A1*, *17βHSD*, and *CYP19A1*) was normalized to the expression of GAPDH. Recombinant resistin at all investigated doses stimulated significantly *CYP11A1* and *3βHSD* expression in SFs (*P* < 0.05). Resistin at 1, 10, and 100 ng/ml also increased *17βHSD* expression in SFs, but no significant changes in expression were observed for *CYP17A1* and *CYP19* (Figure 3A).

Likewise, the expression of *CYP11A1* (at 0.1, 1, 10, and 100 ng/ml of resistin), *3βHSD* (at 0.1, 1, and 10 ng/ml of resistin), *CYP17A1* (at 0.1, 1, 10, and 100 ng/ml of resistin), and *17βHSD* (at 1, 10, and 100 ng/ml of resistin) increased significantly in MFs after the addition of recombinant resistin (Figure 3B, *P* < 0.05). Recombinant resistin had no effect on *CYP19* expression.

Recombinant resistin also up-regulated the expression of *CYP11A1* (at 0.1, 1, 10, and 100 ng/ml of resistin), *3βHSD* (at 1, 10, and 100 ng/ml of resistin), *CYP17A1* (at 100 ng/ml of resistin), and *17βHSD* (at 0.1, 1, 10, and 100 ng/ml of resistin) in LFs (Figure 3C, *P* < 0.05); however, *CYP19* expression in LFs remained unchanged by recombinant resistin.

### Effects of recombinant human resistin on the protein expression of steroidogenic enzymes

**Figure 4:**
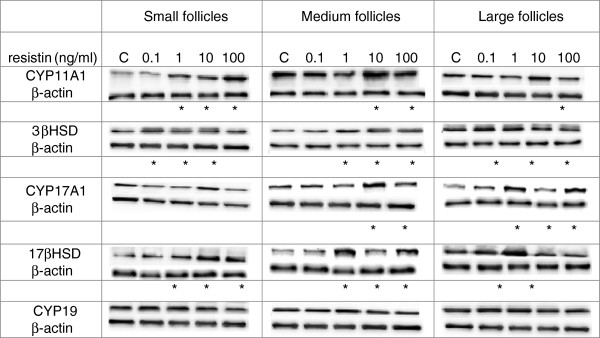
**Effects of recombinant human resistin. (0.1, 1, 10, and 100 ng/ml) on the protein expression of steroidogenic enzymes CYP11A1, 3βHSD, CYP17A1, 17βHSD, and CYP19 small, medium and large ovarian follicles.** The representative samples of western blots are shown in the panels. Western blot experiments were repeated three independent times using samples from three different animals. Data were plotted as mean±S.E.M. Statistical significance is indicated by * *P* < 0.05.

**Figure 5:**
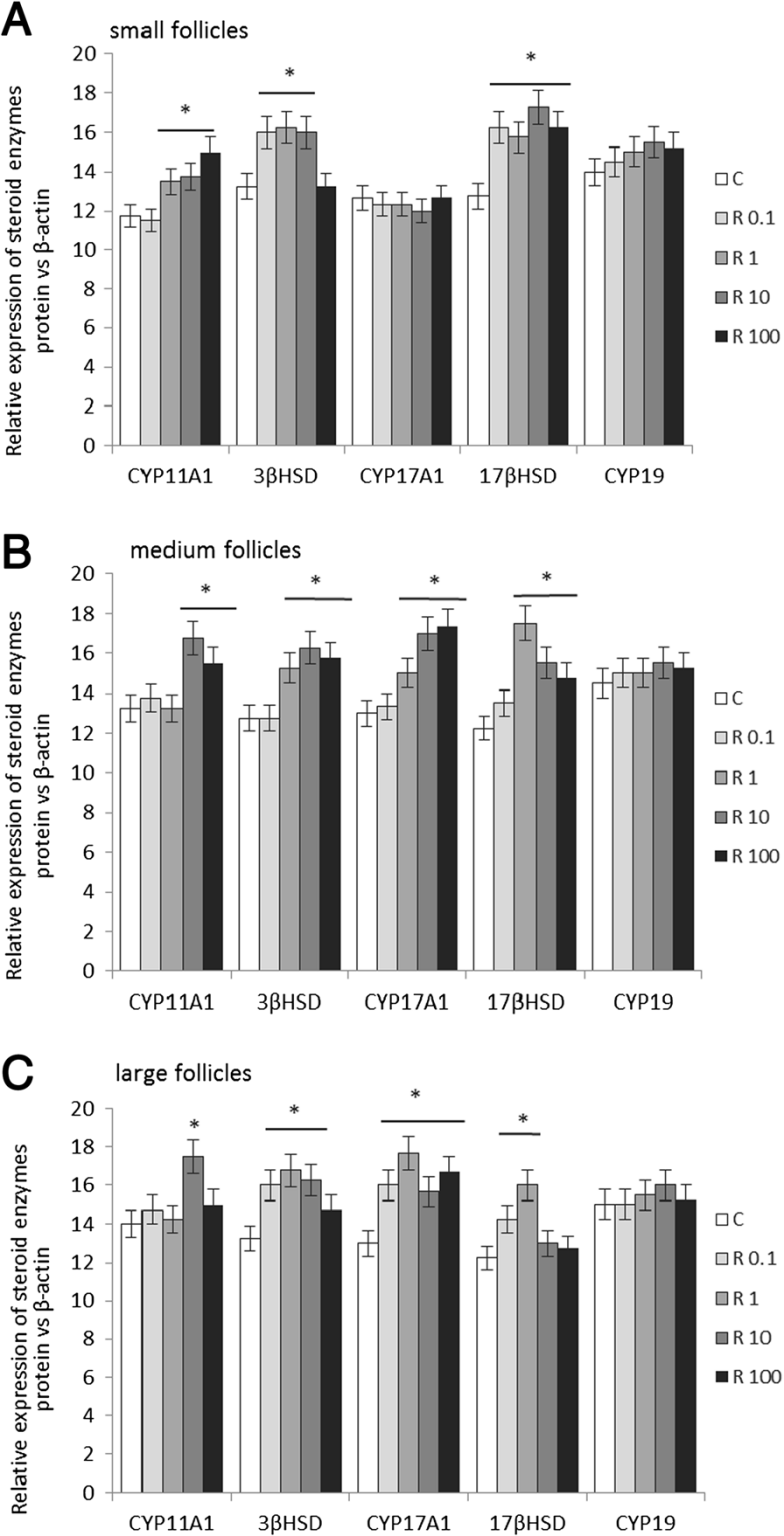
**Densitometric analysis of the protein levels of steroidogenic enzymes CYP11A1, 3βHSD, CYP17A1, 17βHSD, and CYP19 in A) small, B) medium and C) large ovarian follicles.** Proteins were densitometrically scanned and normalized against β-actin. Western blot experiments were repeated three independent times using samples from three different animals. Data were plotted as mean±S.E.M. Statistical significance is indicated by * *P* < 0.05.

The steady state levels of CYP11A1, 3βHSD, CYP17A1, 17βHSD, and CYP19 were investigated in developing ovarian follicles incubated with recombinant resistin, and representative immunoblots from this experiment are shown in Figure 4. Proteins were densitometrically scanned and normalized against β-actin (Figure 5). Resistin at different concentrations up-regulated the expression of CYP11A1, 3βHSD, and 17βHSD in SFs (Figure 5A, *P* < 0.05).

Resistin at increasing concentrations also stimulated CYP11A1, 3βHSD, CYP17A1, and 17βHSD in MFs (Figure 5B, *P* < 0.05).

Significant differences were also observed in the steady state levels of CYP11A1, 3βHSD, CYP17A1, and 17βHSD following incubation of LFs with increasing concentrations of resistin; however, CYP19 remained unchanged by recombinant resistin (Figure 5C).

## Discussion

Female puberty, the estrous cycle, and reproduction are known to be regulated by the hypothalamic-pituitary axis which is comprised of many critical hormones. Many studies have shown that locally produced factors such as steroids, growth factors, and peptides regulate the development of ovarian follicles and the onset of puberty and/or ovulation [23]. Other studies have reported the expression of leptin, an adipose tissue hormone, in the ovary [24], where it was found to regulate ovarian function during puberty [25] and the estrous cycle in humans, mice, rats, pigs, and cows [26-28]. Unfortunately, the role of resistin in ovarian follicle development is currently unknown. The present study reports for the first time the expression of resistin in porcine prepubertal ovarian follicles. Using real time PCR, Western blot, and ELISA, we show differences in the expression and concentration of resistin in follicular fluid collected from SFs, MFs, and LFs. In ovarian follicles collected from prepubertal animals, a ~1.6-fold increase in expression and a ~2-fold increase in protein were observed in LFs, correlating with resistin levels in follicular fluid and follicular tissue. Our results show that resistin expression in the prepubertal ovary also correlated with an increase in estradiol in follicular fluid. Puberty is characterized by increasing concentrations of estradiol, driven by increasing concentrations of pituitary gonadotropins which are, in turn, regulated by gonadotropin-releasing hormone that is released by hypothalamic neurons [29]. Chen et al. [30] used 3 T3-L1 adipocytes to show that estradiol stimulates resistin expression in a dose- and time-dependent manner. This is in agreement with other studies that showed androgens [31] and growth hormones [32] to increase during puberty, and to stimulate the expression of resistin. Additionally, a strong link between resistin and development has been found in rats, where resistin expression was highest at puberty only to decrease in adipose tissue [12]. Moreover, Li et al. [33] showed human resistin levels to decrease with age and puberty in both genders.

In agreement with our results, Maillard et al. [16] showed resistin expression in the bovine and rat ovary. For instance, they demonstrated resistin to be widely expressed in SFs (<6 mm) and LFs (>6 mm), corpora lutea, and oocytes as well as in cumulus, theca, and granulosa cells. Interestingly, resistin expression was approximately 5-fold higher in freshly isolated granulosa cells than in cells that were cultured *in vitro*. Within the ovary, resistin expression was detected in the corpus luteum, oocyte, theca, and granulosa cells [16]. Jones et al. [17] also showed resistin expression in the rat ovary. In an earlier study, Dai et al. [34] cloned and characterized porcine resistin, and they found serum resistin to circulate as a dimer and a trimer and they also suggest that pig may be a suitable animal model for studying the function of resistin in human insulin resistance [34].

The *in vitro* effects of recombinant resistin on steroid hormone secretion and steroid enzyme expression were also interesting as they may represent a unique mechanism of resistin action in the prepubertal ovary. Our results showed that recombinant resistin increased P4, A4, and T secretion in conditioned culture media from MFs and LFs; however, E2 secretion was unaffected. These results were confirmed by additional experiments that showed recombinant resistin to affect the steady state levels of CYP11A1, 3βHSD, CYP17A1, and 17βHSD. These findings are also consistent with a previous *in vitro* study by Munir et al. [19] that showed resistin to increase androgen production in cultured human theca interna tissue from small antral follicles (8–9 mm) normal cycling premenopausal women. They also observed that resistin (1 ng/ml) or a combination of resistin, forskolin, and insulin, enhanced CYP17 expression and 17α-hydroxylase activity (a marker of ovarian hyperandrogenism in PCOS women) [19]. In our study we used porcine whole ovarian follicles collected from prepubertal animals. Prenatal, perinatal, or postnatal height androgen exposure in many species such as in monkeys [35], sheep [36], rats [37], and mice [38] can induce many symptoms characteristic of PCOS. Nevertheless, data on the effects of resistin on ovarian steroidogenesis are contradictory. In cultured rat granulosa cells, P4 secretion increased following treatment with a physiological dose of resistin (10 ng/ml) with no effects on E2, while resistin decreased both P4 and E2 production by granulosa cells isolated from the cow ovary [16]. These contradictory findings led the authors to conclude that resistin is regulated differently across species [16]. Furthermore, Spicer et al. [18] showed that resistin inhibited steroidogenesis in theca and granulosa cells from small (1–5 mm) and large (8–22 mm) cattle follicles.

Taken collectively, our data showed that resistin expression increased during follicular development. We also showed that recombinant resistin stimulated androgen secretion from ovarian follicles by up-regulating the steady state levels of CYP11A1, 3βHSD, CYP17A1, and 17βHSD. Lastly, recombinant resistin had no effects on E2 secretion and CYP19A1 expression in porcine prepubertal ovarian follicles.

## Conclusion

In this study, we demonstrated resistin expression in ovarian follicles obtained from prepubertal pigs. This expression increased during follicular development, with LFs expressing the highest level of the adipokine. We also demonstrated that recombinant resistin affected ovarian steroidogenesis by stimulating androgen production and steroid enzyme expression. In conclusion, the presence of resistin in the porcine ovary and its direct effects on steroidogenesis suggest that resistin may be a new regulator of ovary function in prepubertal animals. However, additional studies on in vivo model are necessary to confirm this hypothesis.

## Abbreviations

A4: Androstendione; CYP11A1: Cytochrome P450, family 11, subfamily A, polypeptide 1; CYP17: Cytochrome P-450c17α; CYP17A1: Cytochrome P450, family 17, subfamily A, polypeptide 1; CYP19: Cytochrome P450 aromatase; CYP19A1: Cytochrome P450, family 19, subfamily A, polypeptide 1; E2: Estradiol; ELISA: Enzyme-linked immunosorbent assay; FSH: Follicle stimulating hormone; GAPDH: Glyceraldehyde-3-phosphate dehydrogenase; 3βHSD: 3β-Hydroxysteroid dehydrogenase; 17βHSD: 17β-Hydroxysteroid dehydrogenase; HSD3B1: Hydroxy-δ-5-steroid dehydrogenase, 3β- and steroid δ-isomerase 1; HSD17B1: Hydroxysteroid (17-β) dehydrogenase 1; LF: Large follicle; LH: Luteinizing hormone; MF: Medium follicle; P4: Progesterone; R: Resistin; SF: Small follicle; T: Testosteron

## Competing interests

The authors declare that they have no competing interests.

## Authors’ contributions

AR-M contributed substantially to the conception and design of this study, performed experiments, and interpreted all data. MD performed the ELISA experiments. ELG drafted and revised the manuscript, evaluating it critically for important intellectual content. All authors read and approved the final manuscript.
